# Supplementary material for: Resolving the full spectrum of human genome variation using Linked-Reads
Source: Genome Res. 2019 Apr;29(4):635–45. doi: 10.1101/gr.234443.118 (PMC6442396; doi:10.1101/gr.234443.118)
Supplement: Supplemental Material [file supp_gr.234443.118_Supplemental_longranger-2.2.zip › longranger-2.2.tar.bz2!/lr2.2-public/lib/stan/cmdstan/stan/doc/api/html/structstan_1_1gm_1_1set__param__ranges__visgen.html]

Stan: stan::gm::set\_param\_ranges\_visgen Struct Reference


|  |  |
| --- | --- |
|  | Stan  2.5.0  probability, sampling & optimization |


- Main Page
- Related Pages
- Namespaces
- Classes
- Files

- Class List
- Class Index
- Class Hierarchy
- Class Members

All Classes Namespaces Files Functions Variables Typedefs Enumerations Enumerator Friends Macros Pages

- stan
- gm
- set\_param\_ranges\_visgen

Public Member Functions |
List of all members

stan::gm::set\_param\_ranges\_visgen Struct Reference

`#include <generator.hpp>`

Inheritance diagram for stan::gm::set\_param\_ranges\_visgen:

|  |  |
| --- | --- |
| Public Member Functions | |
|  | set\_param\_ranges\_visgen (std::ostream &o) |
|  | |
| void | operator() (const nil &) const |
|  | |
| void | operator() (const int\_var\_decl &x) const |
|  | |
| void | operator() (const double\_var\_decl &x) const |
|  | |
| void | operator() (const vector\_var\_decl &x) const |
|  | |
| void | operator() (const row\_vector\_var\_decl &x) const |
|  | |
| void | operator() (const matrix\_var\_decl &x) const |
|  | |
| void | operator() (const unit\_vector\_var\_decl &x) const |
|  | |
| void | operator() (const simplex\_var\_decl &x) const |
|  | |
| void | operator() (const ordered\_var\_decl &x) const |
|  | |
| void | operator() (const positive\_ordered\_var\_decl &x) const |
|  | |
| void | operator() (const cholesky\_factor\_var\_decl &x) const |
|  | |
| void | operator() (const cholesky\_corr\_var\_decl &x) const |
|  | |
| void | operator() (const cov\_matrix\_var\_decl &x) const |
|  | |
| void | operator() (const corr\_matrix\_var\_decl &x) const |
|  | |
| void | generate\_increment\_i (std::vector< expression > dims) const |
|  | |
| void | generate\_increment (std::vector< expression > dims) const |
|  | |
| void | generate\_increment (expression K, std::vector< expression > dims) const |
|  | |
| void | generate\_increment (expression M, expression N, std::vector< expression > dims) const |
|  | |
| Public Member Functions inherited from stan::gm::visgen | |
|  | visgen (std::ostream &o) |
|  | |

|  |  |
| --- | --- |
| Additional Inherited Members | |
| Public Types inherited from stan::gm::visgen | |
| typedef void | result\_type |
|  | |
| Public Attributes inherited from stan::gm::visgen | |
| std::ostream & | o\_ |
|  | |

## Detailed Description

Definition at line 2328 of file generator.hpp.

## Constructor & Destructor Documentation

|  |  |  |  |  |  |
| --- | --- | --- | --- | --- | --- |
| stan::gm::set\_param\_ranges\_visgen::set\_param\_ranges\_visgen | ( | std::ostream & | *o* | ) |  |

inline

Definition at line 2329 of file generator.hpp.

## Member Function Documentation

|  |  |  |  |  |  |
| --- | --- | --- | --- | --- | --- |
| void stan::gm::set\_param\_ranges\_visgen::generate\_increment | ( | std::vector< expression > | *dims* | ) | const |

inline

Definition at line 2468 of file generator.hpp.

|  |  |  |  |
| --- | --- | --- | --- |
| void stan::gm::set\_param\_ranges\_visgen::generate\_increment | ( | expression | *K*, |
|  |  | std::vector< expression > | *dims* |
|  | ) |  | const |

inline

Definition at line 2480 of file generator.hpp.

|  |  |  |  |
| --- | --- | --- | --- |
| void stan::gm::set\_param\_ranges\_visgen::generate\_increment | ( | expression | *M*, |
|  |  | expression | *N*, |
|  |  | std::vector< expression > | *dims* |
|  | ) |  | const |

inline

Definition at line 2491 of file generator.hpp.

|  |  |  |  |  |  |
| --- | --- | --- | --- | --- | --- |
| void stan::gm::set\_param\_ranges\_visgen::generate\_increment\_i | ( | std::vector< expression > | *dims* | ) | const |

inline

Definition at line 2456 of file generator.hpp.

|  |  |  |  |  |  |
| --- | --- | --- | --- | --- | --- |
| void stan::gm::set\_param\_ranges\_visgen::operator() | ( | const nil & |  | ) | const |

inline

Definition at line 2332 of file generator.hpp.

|  |  |  |  |  |  |
| --- | --- | --- | --- | --- | --- |
| void stan::gm::set\_param\_ranges\_visgen::operator() | ( | const int\_var\_decl & | *x* | ) | const |

inline

Definition at line 2333 of file generator.hpp.

|  |  |  |  |  |  |
| --- | --- | --- | --- | --- | --- |
| void stan::gm::set\_param\_ranges\_visgen::operator() | ( | const double\_var\_decl & | *x* | ) | const |

inline

Definition at line 2356 of file generator.hpp.

|  |  |  |  |  |  |
| --- | --- | --- | --- | --- | --- |
| void stan::gm::set\_param\_ranges\_visgen::operator() | ( | const vector\_var\_decl & | *x* | ) | const |

inline

Definition at line 2359 of file generator.hpp.

|  |  |  |  |  |  |
| --- | --- | --- | --- | --- | --- |
| void stan::gm::set\_param\_ranges\_visgen::operator() | ( | const row\_vector\_var\_decl & | *x* | ) | const |

inline

Definition at line 2362 of file generator.hpp.

|  |  |  |  |  |  |
| --- | --- | --- | --- | --- | --- |
| void stan::gm::set\_param\_ranges\_visgen::operator() | ( | const matrix\_var\_decl & | *x* | ) | const |

inline

Definition at line 2365 of file generator.hpp.

|  |  |  |  |  |  |
| --- | --- | --- | --- | --- | --- |
| void stan::gm::set\_param\_ranges\_visgen::operator() | ( | const unit\_vector\_var\_decl & | *x* | ) | const |

inline

Definition at line 2368 of file generator.hpp.

|  |  |  |  |  |  |
| --- | --- | --- | --- | --- | --- |
| void stan::gm::set\_param\_ranges\_visgen::operator() | ( | const simplex\_var\_decl & | *x* | ) | const |

inline

Definition at line 2379 of file generator.hpp.

|  |  |  |  |  |  |
| --- | --- | --- | --- | --- | --- |
| void stan::gm::set\_param\_ranges\_visgen::operator() | ( | const ordered\_var\_decl & | *x* | ) | const |

inline

Definition at line 2390 of file generator.hpp.

|  |  |  |  |  |  |
| --- | --- | --- | --- | --- | --- |
| void stan::gm::set\_param\_ranges\_visgen::operator() | ( | const positive\_ordered\_var\_decl & | *x* | ) | const |

inline

Definition at line 2393 of file generator.hpp.

|  |  |  |  |  |  |
| --- | --- | --- | --- | --- | --- |
| void stan::gm::set\_param\_ranges\_visgen::operator() | ( | const cholesky\_factor\_var\_decl & | *x* | ) | const |

inline

Definition at line 2396 of file generator.hpp.

|  |  |  |  |  |  |
| --- | --- | --- | --- | --- | --- |
| void stan::gm::set\_param\_ranges\_visgen::operator() | ( | const cholesky\_corr\_var\_decl & | *x* | ) | const |

inline

Definition at line 2415 of file generator.hpp.

|  |  |  |  |  |  |
| --- | --- | --- | --- | --- | --- |
| void stan::gm::set\_param\_ranges\_visgen::operator() | ( | const cov\_matrix\_var\_decl & | *x* | ) | const |

inline

Definition at line 2428 of file generator.hpp.

|  |  |  |  |  |  |
| --- | --- | --- | --- | --- | --- |
| void stan::gm::set\_param\_ranges\_visgen::operator() | ( | const corr\_matrix\_var\_decl & | *x* | ) | const |

inline

Definition at line 2443 of file generator.hpp.

---

The documentation for this struct was generated from the following file:

- src/stan/gm/generator.hpp


---

[ Stan Home Page ]

*© 2011–2014,
Stan Development Team.*
